# Assessment of Angular and Straight Linear Rowing Ergometers at Different Intensities of Exercise

**DOI:** 10.3390/s24175686

**Published:** 2024-08-31

**Authors:** Ricardo Cardoso, Manoel Rios, Pedro Fonseca, Joana Leão, Filipa Cardoso, Jose Arturo Abraldes Abraldes, Beatriz B. Gomes, João Paulo Vilas-Boas, Ricardo J. Fernandes

**Affiliations:** 1 Centre of Research, Education, Innovation and Intervention in Sport, Faculty of Sport, University of Porto, 4200-450 Porto, Portugal; manoel.rios@hotmail.com (M.R.); up202005623@edu.fade.up.pt (J.L.); up201402398@edu.fade.up.pt (F.C.); jpvb@fade.up.pt (J.P.V.-B.); ricfer@fade.up.pt (R.J.F.); 2 Porto Biomechanics Laboratory, Faculty of Sport, University of Porto, 4200-450 Porto, Portugal; pedro.labiomep@fade.up.pt; 3 Research Group Movement Sciences and Sport (MS&SPORT), Department of Physical Activity and Sport, Faculty of Sport Sciences, Campus San Javier, University of Murcia, 30720 San Javier, Murcia, Spain; abraldes@um.es; 4 CIDAF—Research Unit for Sport and Physical Activity, Faculty of Sport Sciences and Physical Education, University of Coimbra, 3040-248 Coimbra, Portugal; beatrizgomes@fcdef.uc.pt

**Keywords:** rowing ergometry, biomechanics, physiology, kinematics

## Abstract

We aimed to conduct a biophysical comparison of angular (Biorower) and linear (Concept2) rowing ergometers across a wide spectrum of exercise intensities. Sixteen (eleven male) skilled rowers, aged 29.8 ± 8.6 and 23.6 ± 1.5 years, with international competitive experience, performed 7 × 3 min bouts with 30 W increments and 60 s intervals, plus 1 min of all-out rowing on both machines with 48 h in between. The ventilatory and kinematical variables were measured breath-by-breath using a telemetric portable gas analyzer and determined using a full-body markerless system, respectively. Similar values of oxygen uptake were observed between ergometers across all intensity domains (e.g., 60.36 ± 8.40 vs. 58.14 ± 7.55 mL/min/kg for the Biorower and Concept2 at severe intensity). The rowing rate was higher on the Biorower vs. Concept2 at heavy and severe intensities (27.88 ± 3.22 vs. 25.69 ± 1.99 and 30.63 ± 3.18 vs. 28.94 ± 2.29). Other differences in kinematics were observed across all intensity domains, particularly in the thorax angle at the finish (e.g., 19.44 ± 4.49 vs. 27.51 ± 7.59° for the Biorower compared to Concep2 at heavy intensity), likely due to closer alignment of the Biorower with an on-water rowing technique. The overall perceived effort was lower on the Biorower when compared to the Concept2 (14.38 ± 1.76 vs. 15.88 ± 1.88). Rowers presented similar cardiorespiratory function on both rowing ergometers, while important biomechanical differences were observed, possibly due to the Biorower’s closer alignment with an on-water rowing technique.

## 1. Introduction

Rowing performance is highly influenced by the interplay between the rower’s physiology and biomechanics, as well as by the type of boat and oars used to for training and competition [1]. Since rowing performance is highly affected by weather conditions, rowing ergometers are a versatile tool to conduct training year-round, particularly during the winter season [2,3]. Although there are concerns regarding the discrepancy between the physiological stress and the biomechanical properties of an ergometer vs. on-water rowing, these machines are a common tool for testing rowers’ physiological and kinematic profiles [4,5,6]. However, while they simulate the physiological demands of on-water rowing [7], they might negatively impact rowers’ technique, since the skill requirements are different from rowing on a boat [4].

Rowing ergometers have been used for decades and are accepted as universal, valuable, and standardized instruments for training and testing rowers during their preparatory training phase [8,9,10]. The most common is the stationary linear ergometer, an air-braked device positioned directly on the floor, featuring a sliding seat and a fixed foot stretcher, facilitating the rowing movement [11]. Dynamic ergometers are now available to increase specificity to on-water technical demands, being placed on slides or having a moveable flywheel and foot stretcher. This allows the rower’s center of mass to stay in a nearly fixed position, since the seat does not move the same distance as it does in the stationary ergometer [2,9,11,12]. These ergometers deliver a horizontal linear movement of the handle during the recovery and propulsive phases, since it is attached to a chain that makes the flywheel spin. Concurrent to this kinematic similarity, it is essential that the ergometer adequately simulates the physiological determinants of rowing performance [13], but data comparing both types of rowing ergometers are contradictory (probably due to the use of different protocols and rowers of diverse skill levels [14,15]).

Although not so frequently available, the Biorower is a dynamic rowing ergometer with an angular motion of the handles, built to simulate the actual on-water sculling technique. Instead of using a chain to move the flywheel (as most conventional ergometers do), it has two levers that simulate the angular movement of the sculling oars, aiming to more closely replicate the rowing motion and boat movement. The aim of the current study was to compare angular (with oars) and straight linear rowing ergometers regarding relevant physiological and biomechanical variables across a wide spectrum of exercise intensities. We hypothesized that the angular rowing ergometer would elicit similar cardiorespiratory values at the same rowing intensity, while differences in kinematics (e.g., smaller rowing length and thorax angles at the finish position) would reflect the Biorower alignment with on-water rowing technique. Complementarily, due to the typically high training volume, the cyclic nature of rowing, and the high load on the low back muscles, the risk of injury increases [16,17]. To address this issue, rowers performed a plank test at baseline and post-exercise to quantify the acute effect of each rowing ergometer on core muscles activation. It was expected that the Biorower would present a lower degree of post-exercise core muscle fatigue compared to the stationary ergometer.

## 2. Materials and Methods

Sixteen skilled rowers (eleven males and five females 29.8 ± 8.6 and 23.6 ± 1.5 years of age, with heights of 177.1 ± 5.5 and 168.6 ± 6.8 cm, with body masses of 77.9 ± 6.0 and 66.3 ± 7.2 kg, and body mass indexes of 24.8 ± 1.6 and 23.3 ± 1.8 kg∙m^−2^, respectively), participated in the current study. All of the rowers were recruited according to the following criteria: (i) more than three years of experience in rowing training and competition; (ii) aged between 18 and 40 years old; and (iii) absence of musculoskeletal injuries over the last six months. Subjects were instructed to refrain from hard physical activity and to maintain their individual nutritional habits (avoiding the intake of alcohol, caffeine, and food supplements) 24 h prior to the experiment. The current study was approved by the local research ethics committee (CEFADE 27/2020) and followed the principles established by the Declaration of Helsinki. Participants were informed about the risks and benefits of the study, and signed informed consent forms.

**Figure 1:**
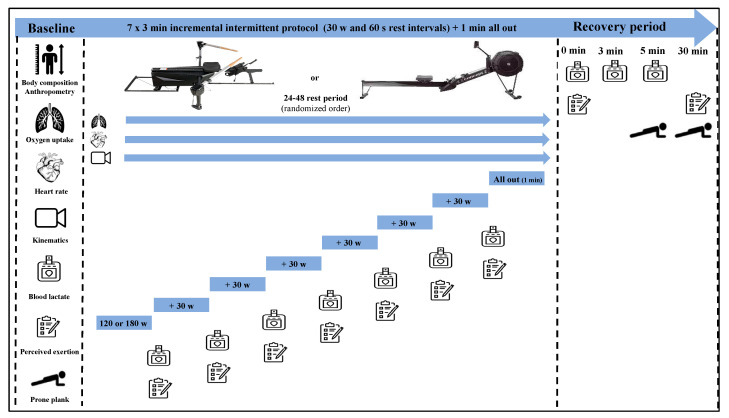
Incremental rowing protocol and post-exercise setup with the data collection time course, displaying the angular and straight linear ergometers.

A randomized design was used to compare the physiological and biomechanical responses to incremental exercise performed on an angular rowing ergometer (with oars) (Biorower S1 Club, Biorower, Vienna, Austria) and a linear rowing ergometer (Model D, Concept2, Morrisville, VT, USA) (Figure 1). Both devices were equipped with real-time feedback monitor screens (Biorower App v 1.071 and PM5, respectively), which displayed the average power and rowing cycle rate. The drag factor was individually set according to feedback from coaches and the rowers’ training habits. Tests were performed in a laboratory facility under the same environmental conditions (22.5 °C ambient temperature and 55% humidity) and were supervised by experienced researchers. Rowers underwent two familiarization sessions on the Biorower (lasting 45 to 60 min each).No adaptation was conducted on the Concept2 since the rowers had significant prior experience.

The experimental protocol performed on both rowing ergometers (with a 48 h interval in between) consisted of 7 × 3 min bouts with 30 W increments (starting at 180 and 120 W for the male and the female participants, respectively), a 60 s rest interval between steps, plus a 1 min all-out effort [3,6,15]. Respiratory and pulmonary gas exchange data were measured breath-by-breath at rest, during exercise, and in the recovery period using a telemetric portable gas analyzer (K5, Cosmed, Rome, Italy) previously calibrated with gases of known concentrations (16% O_2_ and 5% CO_2_) [18,19]. Erroneous breaths were excluded and only data within the mean of ± 3 SD were considered, after which smoothing with a moving average of three breaths and 10 s, respectively, was performed [18,20]. Maximal oxygen uptake was determined using conventional physiological criteria (particularly a plateau despite power increase) and heart rate was measured continuously using a Garmin Edge 830 monitor (Garmin, Olathe, KS, USA), which transmitted telemetrically to the portable gas analyzer [6,21].

To analyze blood lactate concentrations, capillary blood samples (5 μL) were collected from the earlobe at rest, between the steps, immediately after the test, and at the 3rd and 5th min of the recovery, using a Lactate Pro 2 analyzer (Arkay, Inc., Kyoto, Japan) [3,21]. The lactate-power curve was used to assess the anaerobic threshold using the least square method [20,22], allowing determination of the following exercise intensity domains: (i) low and moderate intensities, corresponding to the two steps below and the anaerobic threshold (respectively); (ii) the heavy and severe intensities, matching the steps below and where maximal oxygen uptake was elicited (respectively); and (iii) the extreme intensity, allocated to the 1 min step maximum exertion at the end of the protocol [21,23]. A self-reported rating of perceived exertion was assessed at baseline and at the 30th min of the recovery using the 6 to 20 Borg scale [18,21,24]. A prone plank exercise was used to characterize the muscle function responses at baseline and at 5th and 30th min of the recovery by measuring the time the position was maintained using a chronometer (Seiko, Tokyo, Japan). Rowers had their body weight supported by toes and forearms, with the test ending when they could no longer keep a straight back, lowering the hips [24,25].

Kinematic data were obtained using eight video cameras (Miqus, Qualisys AB, Göteborg, Sweden), operating at a 100 Hz sampling rate and a 720p resolution, with a calibration error of less than 0.50 mm [21]. Full-body markerless kinematics with 6° of freedom were obtained using the Theia Markerless (Theia Markerless, Kingston, ON, Canada) software v2024.03.3 [26] and a 60 s recording (from 60 to 120 s) was performed for each exercise step, while the 1 min final step was recorded entirely. Kinematic analysis of the markerless model was performed in Visual 3D software v2024.06.2 (HAS-Motion, Kingston, ON, Canada) [27] and variables were calculated using a custom-made pipeline. To allow a comparison between ergometers, a midpoint between the hands’ center of mass was created to represent a common handle, because the Biorower allows independent movement of both hands (unlike the Concept2, where both hands hold a single handle).

**Figure 2:**
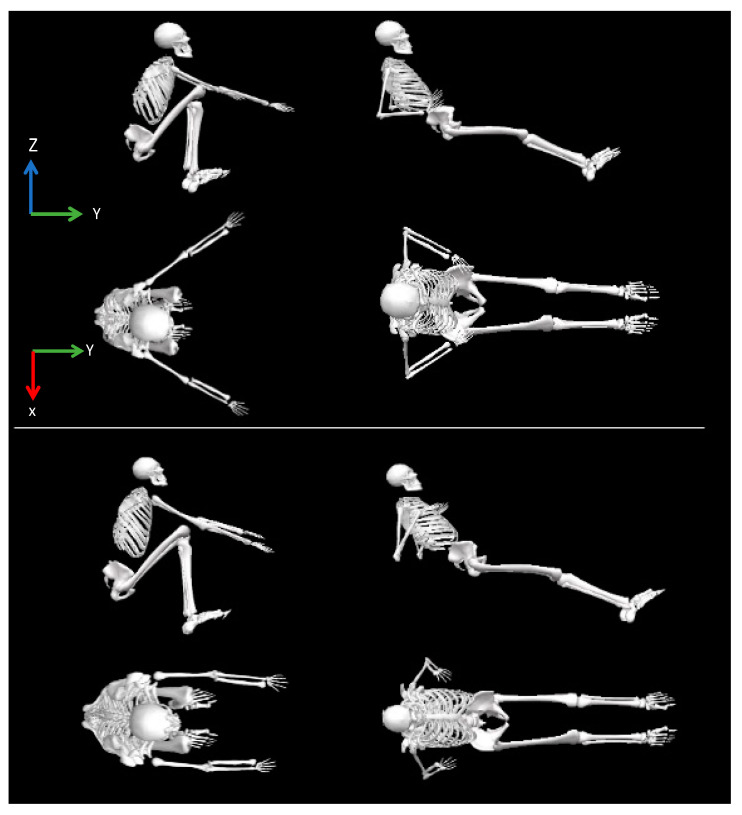
Example of the sagittal and transverse rowing positions on the Biorower and Concept2 ergometers at the catch and finish instants (**left** and **right** panels).

The rowing rate (the mean rowing cycles per min), mean cycle time (mean duration of one complete cycle), propulsive duration and length (mean duration of the propulsive phase and distance travelled by the handle from the catch to the finish positions), and mean of the maximal seat and handle velocities were measured from the catch to the finish instants of the rowing cycle (Figure 2) [21]. The knee-joint angle was calculated between the shank and thigh, while the thorax absolute angle was determined from this segment relative to the global coordinate system. The thorax (positive) and knee angles indicate extension and flexion, respectively, and the rowing ratio was calculated by dividing the propulsion by the recovery durations.

A priori power analysis was conducted using G*Power 3.1.9.7 (Heinrich Heine-Universität, Düsseldorf, Germany) to estimate the minimum sample size required to test the study hypothesis. For a power of 0.8, a large effect size (0.8), and an overall 0.05 significance level, a sample size of 16 participants seemed appropriate. All statistical procedures were performed using GraphPad Prism and SPSS software (version 10.1.1; GraphPad Software, San Diego, CA and version 29.0; SPSS Inc., Chicago, IL, United States) and descriptive statistics were reported for all variables. Normal data distribution was assessed using the Shapiro–Wilk test and expressed in mean ± standard deviation (SD). A repeated measures ANOVA was used to test the differences along the incremental protocol, and a paired samples t-test was employed to compare the studied physiological and biomechanical variables between rowing ergometers. Intraclass correlation coefficients (ICCs) were used to assess the degree of reliability between rowing ergometers (<0.5—poor, 0.5–0.75—moderate, 0.75–0.9—good and >0.9—excellent [28]). The statistical significance was set at 5% and Cohen’s d effect size (ES) was computed to assess the magnitude of changes between experimental conditions (trivial: < 0.2, small: 0.2–0.6, moderate: 0.6–1.2, large: 1.2–2.0, and very large: > 2.0).

## 3. Results

Data obtained during the experiments are displayed in Table 1, with most of the physiological and biomechanical variables increasing concurrently with the rowing intensity for both ergometers. Particularly, an increase in oxygen uptake was observed from low to severe intensity (followed by a decrease at the extreme domain), while heart rate and blood lactate concentration increased across the entire intensity spectrum (all with *p* < 0.001). Complementarily, the kinematic variables values increased with the rise of the power, while the mean cycle time decreased for both rowing ergometers (*p* < 0.001).

The cardiorespiratory variables’ ICC values observed for the two devices indicated a moderate to excellent reliability across the intensity domains, except for poor reliability for the respiratory quotient at heavy and extreme intensities, and blood lactate concentration at heavy intensity. In contrast, the biomechanical variables generally exhibited poor reliability between devices. Exceptions included moderate reliability for rowing rate at severe intensity, mean cycle time at heavy and severe intensities, propulsive phase duration at low and heavy intensities, maximal handle velocity from low to severe intensities, maximal seat velocity at moderate and heavy intensities, and knee angle at catch at heavy intensity, with good reliability observed at severe and extreme intensities.

Similar values between rowing ergometers were found regarding the cardiorespiratory variables’ response at the different exercise intensity domains, but a lower blood lactate concentration was observed for the Biorower vs. the Concept2 (*p* = 0.04, *d* = 0.50) at extreme intensity even if no power differences were displayed at this domain (*p* = 0.44, *d* = 0.13). Meanwhile, a higher rowing rate and a lower mean cycle time was observed for the Biorower vs. the Concept2 ergometers at heavy (*p* = 0.0006, *d* = 0.82 and *p* = 0.003, *d* = 0.60) and severe intensities (*p* = 0.006, *d* = 0.60 and 0.003, *d* = 0.60). The Biorower presented higher propulsive duration (*p* < 0.0001, *d* = 1.0, *d* = 1.35, *d* = 0.84, *d* = 1.41 and *p* = 0.006, *d* = 0.92) and rowing ratio (*p* = 0.0002, *d* = 1.86, *p* < 0.0001, *d* = 2.06, *p* < 0.0001, *d* = 2.19, *p* < 0.0001, *d* = 2.46 and *p* < 0.0001, *d* = 2.5) from low to extreme intensity domains. Furthermore, the Biorower presented lower length values (*p* < 0.0001, *d* = −3.78, *d* = −4.03, *d* = −3.85, *d* = −4.31 and *d* = −1.96) from low to extreme intensities, respectively.

In addition, the Biorower presented lower values of maximal handle (*p* < 0.0001, *d* = 0.86, *p* < 0.0001, *d* = −0.89, *p* < 0.0001, *d* = −0.33, *p* = 0.0004, *d* = −0.91 and *p* < 0.0001, *d* = −1.02, from low to extreme intensities) and seat velocities (*p* = 0.0002, *d* = −1.02, *p* < 0.0001, *d* = −0.77, *p* = 0.001, *d* = −0.56 from low to heavy intensities and *p* = 0.03, *d* = −0.59 for the extreme intensity, respectively). Concurrently, on the Biorower, the thorax angle values at catch were higher from low to severe intensities (*p* = 0.004, *d* = 0.75, *p* < 0.004, *d* = 0.91, *p* = 0.02, *d* = 0.63, respectively) and lower at finish in all intensity domains (*p* < 0.0001, *d* = −1.43, *p* = 0.0006, *d* = −1.21, *p* = 0.0001, *d* = −1.29, *p* < 0.0001, *d* = −1.31 and *p* = 0.009, *d* = −0.82, from low to extreme, respectively) when compared with the Concept2. Higher knee angles values at catch were observed on the Biorower at the low intensity exertion (*p* = 0.03, *d* = 0.63).

**Figure 3:**
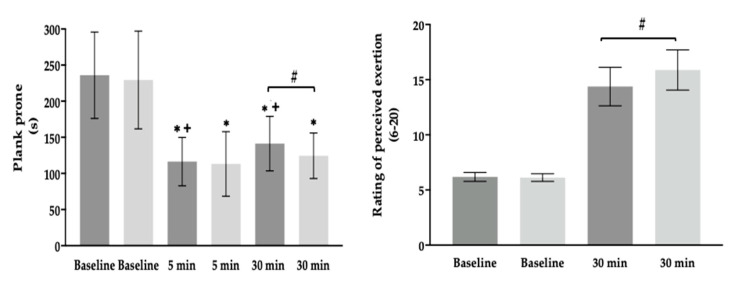
Mean and SD values of the prone plank performance and rating of perceived exertion (**left** and **right** panels) for the Biorower and Concept2 rowing ergometers (dark and light grey). *, ^+^ and ^#^ differences from baseline, 5 min, and 30 min (*p* < 0.05).

The prone plank performance and the rating of perceived exertion values are displayed in Figure 3 (left and right, respectively). Prone plank performance decreased from baseline to 5 min of recovery for Biorower and Concept2 (*p* < 0.0001, *d* = 2.46 and *p* < 0.0001, *d* = 2.02, respectively) and increased from 5 to 30 min on the Biorower (*p* < 0.008, *d* = 0.69). In addition, the Biorower presented less core fatigue (*p* < 0.05, *d* = 0.49) and an overall rating of perceived exertion values (*p* < 0.0001, *d* = 0.82) at the 30 min post-rowing mark, compared with the Concept2.

## 4. Discussion

Rowing ergometers are invaluable tools for enhancing and assessing rowers’ performance, providing a controlled environment free from weather constraints. In addition to the traditional stationary linear rowing ergometer, dynamic devices have recently become available, hypothetically allowing the simulation of the actual angular on-water rowing technique [15]. The data obtained in the current study supported our initial hypothesis since no differences between devices were observed regarding cardiorespiratory variables. However, differences in kinematic variables, such as rowing length and thorax angles at finish and catch positions were found, likely emerging from the mechanical diversity between devices and favouring the Biorower. The higher ICC values observed in the cardiorespiratory variables suggest that these measurements are consistent and reliable across the different intensity domains. This consistency indicates that these variables are not significantly influenced by the devices used. In contrast, the lower ICC values for the biomechanical variables indicate poorer reliability, suggesting that these measurements are more sensitive to the mechanical properties of these rowing ergometers. It is likely that these differences are affecting the mechanical measurements, leading to a reduced reliability between devices.

The use of an intermittent incremental protocol allows for a comprehensive rowing biophysical characterization, facilitating the comparison of both studied ergometers across a wide range of intensity domains [15,21]. Although these machines differ in their mechanical features, resulting in different rowing patterns, similar values of cardiorespiratory function were observed as the exercise intensity increased. These findings align with prior studies comparing dynamic and stationary linear ergometers using incremental protocols [15,29,30]. Given that the Concept2 is considered the gold standard for testing rowers, these results underscore the potential of using the Biorower for indoor assessments with comparable outcomes [31]. While physiological responses between indoor machines and on-water testing are generally similar, subjects might find it more challenging to reach their maximum effort during on-water testing due to the greater technical complexity [32].

The two analyzed rowing ergometers differ substantially in their mechanical structure, leading to distinct rowing movements, especially affecting the upper body. On the Biorower ergometer, the rower must move the mass of the cockpit and power wheel (~100 kg) back and forth on a pair of rails, thus mimicking the angular on-water rowing technique (sculling), while on the Concept2 the rower moves his own body mass (~75 kg) with a linear motion of the handle. The higher rowing rate and the lower mean cycle time on the Biorower at heavy and severe intensities align with studies reporting higher rowing rate in dynamic linear ergometers, applying less force per cycle for a fixed power output [14,33]. In addition, the propulsive phase duration was different between ergometers throughout the intensity domains, suggesting that rowers used different propulsive/recovery ratios to maintain the target power. We also observed that the propulsive phase on the Concept2 ergometer tends to favor maximal handle and seat velocities, benefiting rowers with stronger upper bodies since power is exerted on a single chain [15,34]. Conversely, on the Biorower, the power output is the sum of both oars individually, requiring greater technical proficiency.

Despite mechanical differences between the Biorower and other dynamic linear ergometers previously studied, the reported values in the rowing length corroborate the data from the current study, since the Biorower consistently produced shorter lengths throughout the protocol, possibly attributed to variations in technique between the two ergometers [15,35]. On the Biorower ergometer, the rower must abduct the shoulder horizontally to follow the angular movement of the oar handle, whereas on the Concept2, the rower follows a linear motion, increasing the rowing length (Figure 2). In addition, possibly due to the distinct handle trajectories between ergometers, rowers did not achieve the same “lay back” angle as on the stationary ergometer, instead adopting a closer technique to on-water rowing [17]. From severe to extreme intensity, changes in thorax and knee angles are likely due to the rower’s effort to sustain the rowing length. Furthermore, the changes in thorax angles may be explained by an increased rowing cycle rate and fatigue, which led to greater thoracic flexion as fatigue increases [17,36].

The risk of injury arises from repetition and the transfer of load to the lower back muscles, particularly during prolonged exercise, with the lumbar spine being the most frequently injured region in rowing (2–53%) [16,35,37]. The potential injury risk is supported by the prone plank results, which show that muscle function remains impaired on the Concept2 from 5 to 30 min after exercise compared to the Biorower [24]. Since the core muscles are central to most kinetic chains and transfer forces to the extremities, the rowing (a closed kinetic chain exercise) clearly induces fatigue in this muscle group across different intensity levels [38,39]. Rowers have reported a lower rating of perceived exertion value for the Biorower, likely due to its closer resemblance to on-water rowing, higher technical demands, and possibly due to its lower impact on the lower back muscles.

Some limitations of the current study include the relatively small number of participants, the different mechanical characteristics of these rowing ergometers, particularly in the handles, and the lack of data on power-related variables such as the power profile. Future research should focus on comparing angular ergometers with on-water rowing performance, using standardized race distances and test protocols to address conflicting findings in the literature.

## 5. Conclusions

Rowers exhibited similar cardiorespiratory function on the studied rowing ergometers from low to extreme intensities, while significant biomechanical differences were observed, likely due to the Biorower’s close alignment with the on-water rowing technique. The mechanical differences between rowing ergometers appear to have greater impact on the biomechanical variables, resulting in poor reliability and further highlighting the differences between devices. In addition, post-rowing muscular function showed greater impairment on the Concept2, indicating a need for prolonged recovery compared to the Biorower. This is supported by higher perceived exertion for the Concept2, suggesting that the Biorower may offer potential benefits for injury prevention during extended indoor training. The Biorower simulates on-water rowing, enhancing competition transfer and potentially reducing lower-back stress. In contrast, the Concept2 requires fewer skills but may place greater strain on the lower-back muscles.

## Figures and Tables

**Table 1:** Physiological and biomechanical mean and SD values regarding the two ergometers tested at different intensity domains.

Variables	Low		Moderate		Heavy		Severe		Extreme	
	Biorower	Concept2	Biorower	Concept2	Biorower	Concept2	Biorower	Concept2	Biorower	Concept2
Power (W)	189.13 ± 41.66	193.88 ± 40.69	229.88 ± 48.66	232.81 ± 45.49	266.25 ± 53.24	268.13 ± 47.16	293.00 ± 52.91	296.28 ± 47.83	386.1 ± 75.19	397.6 ± 95.60
Oxygen uptake (mL/min/kg)	43.58 ± 7.03	45.65 ± 6.69	51.60 ± 7.55	50.87 ± 7.26	56.56 ± 9.05	54.39 ± 6.22	60.36 ± 8.40	58.14 ± 7.55	56.30 ± 9.42	53.60 ± 8.38
Respiratory frequency (b/min)	39.89 ± 7.94	40.16 ± 7.94	47.01 ± 8.41	43.28 ± 8.71	52.32 ± 10.78	50.62 ± 9.90	60.11 ± 10.58	57.04 ± 11.27	64.72 ± 15.53	68.02 ± 17.93
Ventilation (L/min)	76.69 ± 18.17	80.39 ± 18.23	100.04 ± 25.23	97.38 ± 21.99	124.75 ± 32.57	119.56 ± 27.34	144.09 ± 32.30	136.62 ± 30.69	146 ± 36.57	147.73 ± 37.22
Respiratory quotient	0.88 ± 0.10	0.91 ± 0.13	0.94 ± 0.10	0.98 ± 0.13	1.03 ± 0.08	1.06 ± 0.14	1.10 ± 0.12	1.12 ± 0.12	1.02 ± 0.10	1.09 ± 0.16
Heart rate (bpm)	151.47 ± 14.08	155.83 ± 14.46	166.64 ± 14.81	168.30 ± 14.56	177.42 ± 11.96	177.32 ± 15.23	183.15 ± 11.83	183.21 ± 13.70	184.39 ± 13.73	183.15 ± 12.98
Blood lactate (mmol/L)	1.97 ± 0.58	1.84 ± 0.58	2.83 ± 0.85	2.83 ± 0.92	4.46 ± 0.95	4.97 ± 1.85	7.41 ± 1.85	7.66 ± 3.02	11.49 ± 3.76	13.35 ± 3.60 *
Rowing rate (cycles/min)	20.63 ± 2.43	20.44 ± 1.93	24.00 ± 2.53	23.06 ± 2.05	27.88 ± 3.22	25.69 ± 1.99 *	30.63 ± 3.18	28.94 ± 2.29 *	42.38 ± 4.78	41.38 ± 4.99
Mean cycle time (s)	2.88 ± 0.31	2.88 ± 0.26	2.51 ± 0.29	2.60 ± 0.22	2.15 ± 0.25	2.28 ± 0.17 *	1.94 ± 0.20	2.03 ± 0.15 *	1.34 ± 0.13	1.40 ± 0.13
Propulsive phase duration (s)	1.17 ± 013	1.04 ± 0.13 *	1.10 ± 0.11	0.97 ± 0.08 *	1.02 ± 0.09	0.95 ± 0.10 *	0.97 ± 0.08	0.87 ± 0.06 *	0.76 ± 0.06	0.70 ± 0.70 *
Rowing ratio	0.70 ± 0.09	0.55 ± 0.07 *	0.80 ± 0.11	0.61 ± 0.07 *	0.94 ± 0.14	0.69 ± 0.08 *	1.05 ± 0.14	0.76 ± 0.09 *	1.31 ± 0.16	1.0 ± 0.07 *
Propulsive phase length (m)	1.18 ± 0.05	1.42 ± 0.07 *	1.19 ± 0.04	1.42 ± 0.07 *	1.19 ± 0.04	1.41 ± 0.06 *	1.18 ± 0.04	1.40 ± 0.06 *	1.06 ± 0.09	1.21 ± 0.06 *
Maximal handle velocity (m/s)	1.68 ± 0.15	1.81 ± 0.15 *	1.77 ± 0.14	1.90 ± 0.15 *	1.85 ± 0.15	1.98 ± 0.14 *	1.91 ± 0.18	2.04 ± 0.12 *	1.99 ± 0.15	2.16 ± 0.18 *
Maximal seat velocity (m/s)	1.00 ± 0.16	1.16 ± 0.15 *	1.10 ± 0.14	1.22 ± 0.13 *	1.19 ± 0.17	1.28 ± 0.13 *	1.26 ± 0.15	1.23 ± 0.31	1.31 ± 0.12	1.25 ± 0.08 *
Thorax angle at catch (°)	−29.32 ± 4.5	−25.66 ± 5.18 *	−30.29 ± 3.01	−26.76 ± 4.57 *	−29.84 ± 4.71	−27.34 ± 5.61	−31.52 ± 5.74	−27.89 ± 5.75 *	−34.94 ± 5.29	−34.01 ± 5.85
Thorax angle at finish (°)	19.53 ± 3.78	27.46 ± 6.87 *	19.88 ± 4.53	27.19 ± 7.18 *	19.44 ± 4.49	27.51 ± 7.59 *	18.30 ± 5.63	26.57 ± 6.86 *	10.66 ± 5.00	15.66 ± 7.09 *
Knee angle at catch (°)	132.50 ± 7.14	136.51 ± 5.37 *	132.00 ± 7.46	135.20 ± 7.12	133.70 ± 7.45	136.10 ± 7.45	134.10 ± 7.39	134.61 ± 6.76	118.30 ± 10.28	117.70 ± 20.23

* Different from Biorower (*p* ≤ 0.05). ICC: <0.5—poor (light grey), 0.5–0.75—moderate (dark grey), 0.75–0.9—good (light blue) and > 0.9—excellent (dark blue).

## Data Availability

Data are contained within the article.
